# WNT and VEGF/PDGF signaling regulate self-renewal in primitive mesenchymal stem cells

**DOI:** 10.21203/rs.3.rs-2512048/v1

**Published:** 2023-04-10

**Authors:** Matteo Mazzella, Keegan Walker, Christina Cormier, Michael Kapanowski, Albi Ishmakej, Azeem Saifee, Yashvardhan Govind, G. Rasul Chaudhry

**Affiliations:** Oakland University; Oakland University; Oakland University; Oakland University; Oakland University; Oakland University; Oakland University; Oakland University

**Keywords:** Mesenchymal stem cells, self-renewal, differentiation, signaling pathway, WNT, VEGF, PDGF, TGFβ, proliferation, senescence

## Abstract

**Background:**

Therapeutic use of multipotent mesenchymal stem cells (MSCs) is hampered due to poor growth and limited self-renewal potential. The self-renewal potential of MSCs is also affected during propagation and changes are poorly understood. This study investigated the molecular mechanism involved in the self-renewal of primitive (p) MSCs.

**Methods:**

pMSCs were cultured to low passage (LP), P3, and high passage (HP), P20, in fetal bovine serum medium (FM) and xeno-free medium (XM). The characteristics of LP and HP pMSCs were evaluated for morphology, expression of cell surface markers, doubling time (DT), colony forming efficiency (CFE), proliferation by BrdU assay, telomerase activity and trilineage differentiation. We then examined transcriptome and nucleosome occupancies using RNA-seq and MNase-seq, respectively analyses.

**Results:**

pMSCs grown in FM gradually changed morphology to large elongated cells and showed a significant reduction in the expression of CD90 and CD49f, CFE, proliferation, and telomerase activity. In addition, cells had a greater propensity to differentiate into the adipogenic lineage. In contrast, pMSCs grown in XM maintained small fibroblastoid morphology, self-renewal, and differentiation potential. Transcriptomic analysis showed upregulation of genes involved in self-renewal, cell cycle, and DNA replication in XM-grown pMSCs. Whereas senescence genes were upregulated in cells in FM. MNase-seq analysis revealed less nucleosomal occupancies in self-renewal genes and senescence genes in pMSCs grown in XM and FM, respectively. The expression of selected genes associated with self-renewal, cell cycle, DNA replication, differentiation, and senescence was confirmed by qRT-PCR. These results led us to propose signaling pathways involved in the self-renewal and senescence of pMSCs.

**Conclusion:**

We conclude that the self-renewal potential of pMSCs is controlled by WNT and VEGF/PDGF, but TGFβ and PI3K signaling induce senescence.

## Background

Mesenchymal stem cells (MSCs) are multipotent stem cells that can be derived from various adult and perinatal sources. The isolated MSCs are characterized based on the criteria established by the Mesenchymal and Tissue Stem Cell Committee of the International Society for Cellular Therapy (1, 2). MSCs are adherent cells with fibroblastoid morphology. They express specific cluster of differentiation (CD) markers such as CD73, CD90, and CD105 and can differentiate into chondrogenic, osteogenic, and adipogenic lineages (3–5). Recently, they have also been differentiated into other lineages, such as neural stem cells, retinal progenitor cells, and myocytes (6–8). In addition, MSCs exhibit anti-inflammatory and immunomodulation effects and home to the site of injury (9). They are preferable to embryonic stem cells (ESCs), which have ethical concerns and can cause teratomas (10, 11). However, unlike ESCs, MSCs grow poorly and gradually lose self-renewal capability upon passaging, particularly in the fetal bovine serum medium (FM) (12). However, the composition of FM varies due to different techniques used to extract the serum and may contain unknown xenogeneic contaminants (13, 14). Several reports describe xeno-free medium (XM) used to culture MSCs (15–19). The XM shows more promise in maintaining the self-renewability of MSCs than FM (20–22). Despite the problems with maintaining MSCs during propagation, molecular changes limiting the self-renewal and differentiation potential upon passaging or culturing are poorly understood. In addition, preclinical and clinical studies performed using MSCs provide inconsistent results partly due to the variability in MSCs source, media, and culture conditions (4, 23). Studies have shown that passaging induces senescence in MSCs (24, 25), but the mechanism of senescence induction is poorly understood. If the molecular changes and associated mechanisms are fully understood, maintaining the self-renewal capacity of MSCs may be possible, and the reproducibility of results using MSCs could be improved. Therefore, we aimed to investigate the molecular changes during in vitro expansion in highly proliferative primitive (p) MSCs isolated from the human umbilical cord (4). Our study showed upregulation of WNT, cell cycle, and DNA replication genes which helps in maintaining the self-renewal and differentiation potential of pMSCs in XM. In contrast, upregulation of TGFβ promoted senescence in the cells in FM. These findings are likely to help expand pMSCs without changing their properties and may have implications in their therapeutic and pharmacological applications.

## Materials And Methods

### Culturing of pMSCs

In this study, we used well-characterized umbilical cord MSCs as described previously (5). Umbilical cord tissue was obtained from consented healthy donors through the Ascension Providence Hospital, Southfield, MI, under an IAA (IAA #400244-10) approved protocol. These cells were maintained by passaging in FM (DMEM nutrient mix F12 medium, Life Technologies, Carlsbad, CA, USA) supplemented with 10% fetal bovine serum (VWR, Radnor, PA, USA), and 5.6% antibiotic solution containing 0.1% gentamicin, 0.2% streptomycin, and 0.12% penicillin (Sigma, St Louis, MO, USA) and XM (MSC NutriStem^®^ XM, Sartorius, Goettingen, Germany) supplemented with MSC NutriStem^®^ XF Supplement Mix (Sartorius, Goettingen, Germany) and 5.6% of the antibiotic solution. pMSCs grown for 3 passages (P) were regarded as low passage (LP) cells, and those passaged to P20 were considered as high passage (HP) cells. LP and HP pMSCs were cultured (1x10^4^/cm^2^) in FM and XM to 70% confluency in a CO_2_ incubator at 37°C.

### Immunophenotyping

Cell surface markers were analyzed using Flow cytometry. LP and HP pMSCs were cultured in their respective medium until 70% confluency. They were then trypsinized and pelleted for analysis. The cells were stained with FITC-conjugated antibodies against CD44, CD49f, and CD90 or APC-conjugated antibodies against CD29, CD73, and CD105 singularly or dually labeled. The cells were then analyzed on a FACS Canto II (Becton Dickinson) using Diva Software (Beckton Dickinson).

### Colony Forming Efficiency (CFE) Assay

LP and HP pMSCs were cultured in FM and XM at a concentration of 100 cells in a 100 mm petri dish. The cells were washed with PBS and fixed after 10-14 days with paraformaldehyde for 10 minutes. Once the cells were fixed, they were stained with 0.1% crystal violet (Thermo Scientific) for 1 hour and rinsed with tap water. Cells were classified as colonies if they had a minimum count of 50 cells. The data was reported as the total colony number per number of plated cells in triplicate.

### Cellular Proliferation Assay

LP and HP pMSCs were suspended in FM and XM and plated at a concentration of 6.25x10^3^ cells/well in a 96-well plate for 2 days. Following the manufacturer’s guidelines, proliferation was assayed using the BrdU proliferation kit in triplicate (Novus Biologicals, Centennial, CO, USA). BrdU assay is widely used to evaluate changes in proliferation due to it being incorporated into newly synthesized DNA.

### Telomerase Assay

Telomerase activity was determined using the telomere repeat amplification protocol (TRAP) assay (26–28). Briefly, whole cell lysates were prepared from cultured cell pellets and lysed in cold RIPA buffer (Santa Cruz) containing protease inhibitor cocktail and sodium orthovanadate at a ratio of 100 μL of buffer per 1.0 x 10^6^ cells. Lysates were cleared by centrifugation at 14,000 rpm for 10 min at 4°C and stored at −80°C. Protein concentration was determined using nanodrop 8000 (Thermofisher).

The SYBR green master mix (Promega) included all components for the quantitative reverse transcriptase-polymerase chain reaction (qRT-PCR). Each well contained 0.1 μg protein lysate, 50% volume SsoAdvanced SYBR green master mix (Bio-Rad), 0.1 μg of each primer TS (5′-AATCCGTCGAGCAGAGTT-3′), and ACX (5′-GCGCGG(CTTACC)3CTAACC-3′) (Integrated DNA Technologies), and RNase/DNase-free water to achieve a final well volume of 25 μL. The qRT-PCR and detection were performed on a CFX 90 (Biorad). In addition to the treatment samples, a series of controls were included on each plate: (1) no template control with TS primer only, (2) no template control with ACX primer only, (3) no template control with TS and ACX primers (used in the normalization of samples), (4) heat-inactivated control with the template (protein lysate) and TS and ACX primers, and (5) HEK cell lysate with TS and ACX primers (a positive control).

### Trilineage Differentiation and Characterization

LP and HP pMSCs were cultured in FM and XM. Twenty-four hours after plating the pMSC, the differentiation was induced using specific differentiation media for 3 weeks (4). The adipogenic differentiation medium comprised of a DMEM nutrient mix F12 containing 0.5 μM isobutyl-methy-lxanthine, 1 μM dexamethasone, 10 μM insulin, and 200 μM indomethacin. The chondrogenic differentiation medium composed of DMEM nutrient mix F12 containing 20ng TGFpi, 10 ng insulin, 100 nM dexamethasone, and 100 μM ascorbic acid. The osteogenic differentiation consisted of DMEM nutrient mix F12 containing 0.1 μM dexamethasone, 10 μM β-glycerophosphate, and 50 μM ascorbate-phosphate. Control pMSCs were cultured using FM or XM.

The differentiated cells for characterized using oil red o, toluidine blue, and alizarin red for adipogenic, chondrogenic and osteogenic differentiation, respectively as previously reported (4). To determine the expression of specific proteins in the differentiated derivatives of pMSCs, they were fixed on coverslips by treating with 4% paraformaldehyde for 10 minutes at room temperature. The fixed cells were permeabilized with 0.5% Triton X-100 (Sigma), blocked in 2% bovine serum albumin (Sigma) in PBS for 1 hour, and subjected to specific primary antibodies at 1:100 dilutions at 4°C overnight. They were then treated with a secondary antibody at 1:200 dilution for 2 hours at room temperature, counterstained with DAPI at 1:200 dilutions for 30 minutes at room temperature, and mounted onto a slide. Fluorescent images were captured using a confocal microscope (NIKON Instruments Inc.). The images were then quantified using ImageJ.

### RNA-Sequencing (RNA-seq)

The total RNA from LP and HP pMSCs cultured in FM and XM were isolated and sequenced by following the previously published protocol (29). Briefly, the RNA was quantified and qualified using Agilent2100 Bioanalyzer (Agilent Technologies, Palo Alto, CA, USA) and Qubit Assay (Life Technologies). RNA with a RNA integrity number (RIN) of 10 was used. KAPA RNA HyperPrep Kit with RiboErase was used to prepare the cDNA libraries following the manufacturer’s protocol and sent for RNA-seq transcriptome analysis.

GENEWIZ (South Plainfield, NJ, USA) performed 2 × 150 bp paired-end read sequencing on the Illumina NovaSeq/HiSeq. An average of 47 million reads was obtained for each sample. Fragments were mapped to reference human genome assembly hg38, and differential gene expression analysis was performed using the Galaxy platform (https://usegalaxy.org/) (30), as detailed below. RNA-Seq analyses were performed on three independent biological replicates.

### Micrococcal Nuclease Sequencing (MNase-seq)

Nucleosomes were cross-linked by formaldehyde and were then lysed. The DNA was then fragmented using MNase digestion and processed using Illumina NEBNext Ultra II Library prep kit for the preparation of the library. The DNA libraries were gel purified and sequenced using Illumina HiSeq-4K in paired-end mode at the University of Michigan Advanced Genomics Core Facility in Ann Arbor, MI, USA. The paired-end reads were mapped to the human genome assembly hg38 and analyzed using the Galaxy platform. MNase-Seq analyses were performed on three independent biological replicates.

### Bioinformatic Analysis

For RNAseq, the FastQC toolkit determined the quality of the raw reads; Trim Galore! was then used to trim low-quality reads and adapters. The raw reads were mapped using the hg38 human genome using HiSat2. FeatureCounts assessed the expression of each gene. DESeq2 was used to normalize the raw counts, and the expression was determined for the differential genes. The genes with an adjusted p-value of < 0.05 and a 1.5 fold for log2(FC) were considered significant. The differential genes were analyzed using protein analysis through evolutionary relationships (PANTHER) and the Kyoto Encyclopedia of Genes and Genomes (KEGG) pathway. Heatmaps were generated to assess expression using Heatmapper (http://www.heatmapper.ca/).

For MNase seq, the adapters sequences were isolated to the genomic inserts at the 5’ to 3’ ends of the reads for analysis by Trim Galore!. Using Bowtie2, the isolated regions are mapped to the hg38 human genome and aligned to their respective locations. The bowtie2 outputs are then merged with Merge SAM/BAM files. The Scale Factor is computed by taking the sum of the reads for each condition and dividing it by the highest number of reads. The data is then normalized by bamCovarage. Use the UCSC Main Genome Browser to analyze transcripts. Afterward, import the files into the ComputeMatrix tool to create a matrix file. The data was then visualized using plotHeatmap and Interactive Genomics Viewer (IGV).

### qRT-PCR

LP and HP pMSC grown in FM and XM were harvested and subjected to the isolation of their total cellular mRNA using the GeneJET RNA purification Kit (Thermo Scientific), following the manufacturer’s instructions. The RNA was purified using a thermocycler (Bio-Rad) and the cDNA was synthesized using the iScript kit (Bio-Rad, Hercules, CA, USA). qRT-PCR was performed using SsoAdvanced SYBR Green Supermix (Bio-Rad) and the CFX90 Real-Time PCR system. The primers (IDT Technologies, Coralville, IA, USA) used for trilineage differentiation, self-renewal, cell cycle, DNA replication, and senescence in this study are in Additional file 1. The reactions were normalized using the reference genes GAPDH and β-ACTIN and were performed in triplicate.

### Statistical Analysis

Data are presented as the mean ± standard error of the mean (SEM) of triplicates per analysis. Results with **p ≤ 0.01 and *p ≤ 0.05 were considered statistically significant. All analyses were performed using SPSS version 26 (SPSS Inc. USA) using the one-way ANOVA test.

## Results

### Characteristics pMSCs grown in FM and XM

**Figure 1:**
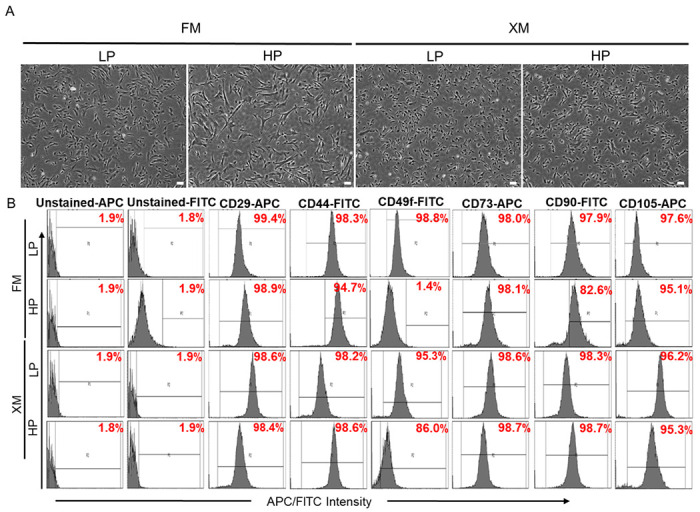
Characteristics of pMSCs at LP and HP in FM and XM. (a-b) Phase contrast microscopy images and expression of surface markers determined by flow cytometer in pMSCs, respectively. Scale bars represent 100 μm (magnification: 4x). HP pMSCs in FM had larger cell size and significantly downregulated expression of CD49f and CD90 compared to LP pMSCs.

Many reports indicate that MSCs progressively lose self-renewal and differentiation potential upon passaging (24, 25). Our results showed that when pMSCs were repeatedly passaged using FM, they became increasingly larger and elongated morphology (Fig. 1a). In contrast, the XM maintained smaller fibroblastoid morphology even after twenty passages. FACS analysis of the LP and HP pMSCs showed maintenance of MSC-specific surface makers, CD29, CD44, CD49f, CD73, CD90, and CD105 in LP and HP pMSCs cultured in XM. While the expression of these markers in LP MSCs cultured in FM and XM was similar, the expression of CD49f and CD90 was significantly reduced in HP MSCs grown in FM (Fig. 1b and Additional file 2).

**Figure 2:**
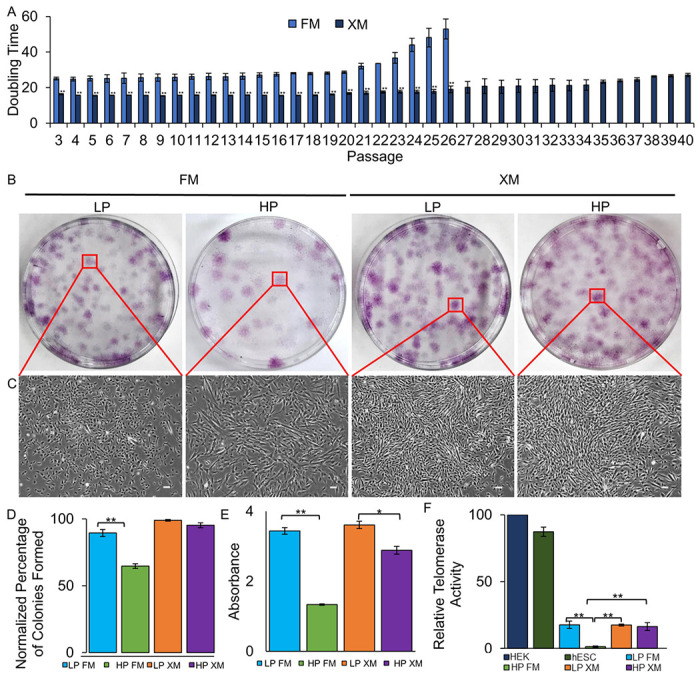
Proliferation and colony-forming efficiency assays of LP and HP pMSCs grown in FM and XM. (a) Doubling time of pMSCs (b-c) Crystal violet stained colonies of pMSCs and phase contrast showing the morphology of a single colony of pMSCs. Scale bars represent 100 μm (magnification: 4x). (d): Percentage of colony formation of LP and HP pMSCs. E: Proliferation of pMSCs as determined using BrdU proliferation kit. (f): Relative telomerase activity in various cell types as determined using the qRT-PCR-based TRAP assay. All values are reported as telomerase activity relative to HEK cells. Growth of pMSCs in FM yielded cells with significantly reduced doubling time and CFE, less compact colonies, and decreased proliferation rate and relative telomerase activity.

XM also maintained a low doubling time (DT) even up to P40, but the DT was gradually and rapidly increased in FM (Fig. 2a). The cell practically stopped growing soon after P25. Similar to the MSC-markers and DT, the clonogenicity of pMSCs was maintained at a high level, 99% and 95% CFE in LP and HP pMSCs, respectively, in XM, but it was significantly reduced, 89% and 65% CFE in LP and HP pMSCs, respectively FM (Fig. 2b–d). Furthermore, cell proliferation assay demonstrated higher BrdU uptake (OD, 3.6 and 2.9) in LP and HP pMSCs, respectively, grown in XM compared to LP and HP pMSCs (OD, 3.4 and 1.3, respectively) grown in FM (Fig. 2e). In addition, the low level telomerase activity was maintained to near LP pMSCs (17.5%) in HP cells (16.4%) grown in XM. Still, it was a significant decrease (1.3%) in HP pMSCs in FM relative to the telomerase activity in HEK cells (100%) and human ESCs (87.2%) (Fig. 2f). Taken together, the rate of proliferation, CFE, and expression of CD90 and CD49f were significantly reduced in pMSCs upon passaging in FM. However, these characteristics were well maintained in XM-grown cells, indicating the gene expression does not significantly change between LP and HP, when grown in XM.

### Trilineage differentiation potential of pMSCs

**Figure 3:**
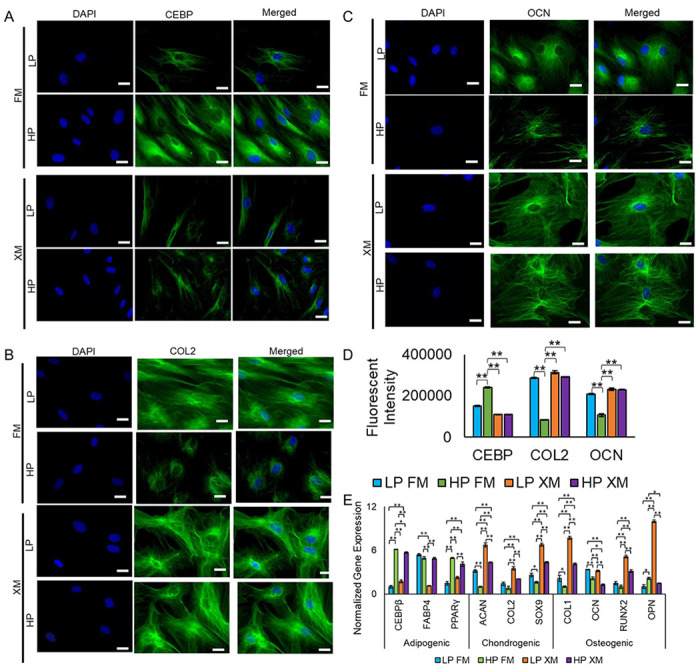
Determination of differentiation potential of LP and HP pMSCs grown in FM and XM. (a-c) Fluorescence microscopic images and fluorescence intensity of the derivatives stained with CEBR COL2, and OCN antibodies representing chondrogenic, osteogenic, and adipogenic lineages, respectively. (d) Quantification of fluorescent intensity of immunostained derivatives of pMSCs. Scale bars represent 400 μm (magnification: 40x). (e) Expression of adipogenic (CEBPβ, FABP4, and PPARγ), chondrogenic (SOX9, ACAN, and COL2), and osteogenic (COL1, RUNX2, OPN, and OCN) genes in the differentiated derivatives. Gene expression was normalized to GAPDH and ACTIN, and error bars represent the standard deviations of the triplicate measurements. HP pMSCs grown in FM showed a greater propensity to differentiate towards adipogenic lineage.

LP and HP pMSCs grown in FM and XM differentiated into chondrogenic, osteogenic, and adipogenic lineages based on the staining of cells with toluidine blue, alizarin red, and oil red o, respectively, as previously described (4). The differentiated derivatives expressed adipogenic, chondrogenic, and osteogenic proteins, CEBP, COL2, and OCN, respectively, as determined by immunostaining (Fig. 3a–c). Interestingly, adipogenic derivatives of HP pMSCs cultured in FM showed a significant increase in the expression of CEBP compared to the cells grown in XM. Whereas chondrogenic and osteogenic derivatives of LP and HP pMSCs cultured in XM and LP pMSCs cultured in FM had a significant higher expression of COL2 and OCN, respectively compared to the derivatives of the cells grown in in FM (Fig. 3d). Overall, pMSCs cultured in the XM showed a greater tendency to differentiate toward the chondrogenic and osteogenic lineage, whereas HP pMSCs cultured in the FM showed a greater tendency to differentiate toward the adipogenic lineage. qRT-PCR analysis of the differentiated derivatives of HP pMSCs cultured in FM had an upregulation of adipogenic genes (*CEPβ, FABP4, and PPARγ*) while those of LP pMSCs cultured in XM had higher expression for chondrogenic (*ACAN, COL2, and SOX9*) and osteogenic (*COL1, OCN, RUNX2, and OPN*) genes (Fig. 3e).

### Differential gene expression

**Figure 4:**
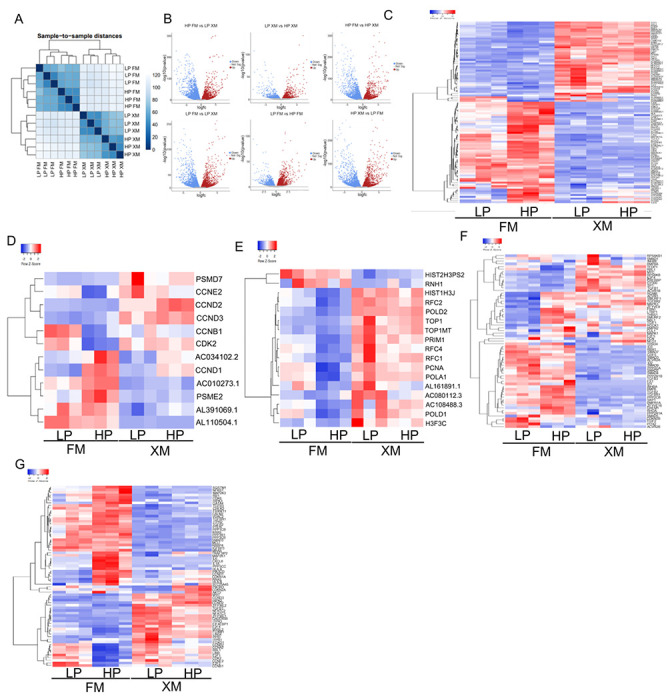
Transcriptional analysis in LP and HP pMSCs grown in FM and XM. (a) Expression heat map of sample-to-sample distances on the matrix of variance-stabilized data for overall gene expression. Darker blue colors indicate a similar correlation (the color key is in arbitrary units). Clustering indicates that pMSCs cultured in XM were similar. Likewise, pMSCs cultured in FM were similar but different from cells grown in XM. (b) Volcano plots showing raw z-scores of RNA-seq log2 transformed values of the DEGs. (c-g) RNA-seq results showing the Wnt signaling, cell cycle, DNA replication, TGF beta, and senescence genes, respectively. Up- and down-regulated genes are red and blue, respectively. HP pMSCs grown in FM displayed down-regulation of Wnt signaling, cell cycle, and DNA replication genes but upregulation of TGF beta and senescence genes.

Based on the analysis of cell morphology, DT, CFE, telomerase activity, and differentiation of LP and HP pMSCs cultured in FM and XM, we envisioned changes in the gene expression. Therefore, we first analyzed the overall gene expression in pMSCs grown in two different media. This analysis showed distinct differences in cells grown in FM and XM (Fig. 4a). Evidently, the cells grown in XM had similar gene expression with slight differences between LP and HP pMSCs. Likewise, cells grown in FM had similar gene expression with slight differences between LP and HP pMSCs. However, there were significant differences between the cells grown in FM and XM.

Next, we compared the differentially expressed genes (DEGs) between the various groups using volcano plots (Fig. 3B). The number of DEGs between the pMSCs grown in FM and XM were significantly more compared to the cells grown in the same medium, irrespective of the passage. The least DEGs were observed between the LP and HP cells grown in XM.

Venn diagram also revealed that most DEGs (749) were found between HP cells grown in FM vs LP cells grown in XM followed by 403 DEGS in LP cells grown in FM vs HP cells grown in XF. In other groups, the difference in DEGs was less than 200. Only 21 DEGs between LP and HP cells were grown in XM (Additional File 3a). This suggests that pMSCs grown XM were more similar compared to cells grown in FM.

Heat map data in Fig. 4c–g show the DEGs related to Wnt signaling, cell cycle, DNA replication, TGFγ, and senescence pathways in pMSCs. The results revealed that many WNT signaling pathway genes were upregulated in pMSCs grown in XM but downregulated in FM (Fig. 4c and Additional File 3b).

The prominent upregulated WNT pathway genes that may positively affect cell proliferation included *DVL2, CSKNK1G2, AXIN1, SRCAP, PRKCD, FZD1, MYC, DVL1, CREBBP*, and *TLE3*. In addition, several genes of SNF/SWI family (*SMARCA4, SMARCD1, SMARCA2*, and *SMARCC2*) involved in chromatin modeling and two genes (*EP300* and *EP400*) associated with histone acetylation were upregulated in XM cells. In contrast, several WNT genes (*PPP2B, SFRP1, TCF7L1, TLE4, PPP2CA, CCND1, PPP2R5B*, and *FZD2*) that may be negatively affecting cell proliferation was upregulated in cells grown in FM but downregulated in XM. In addition, two genes (*TGFBR1* and *BMPR1A*) and one gene (*SMARCA1*) associated with differentiation and chromatin remodeling, respectively, were also upregulated in cells grown in FM. Importantly, the expression of these genes was more significant in HP than LP pMSCs, suggesting that FM-grown cells had reduced self-renewal capacity or undergoing differentiation.

Furthermore, LP and HP pMSCs cultured in XM showed upregulation of cell cycle genes (*PSMD7, CCNE2, CCND2*, and *CDK2*) related to proliferation. In LP and HP pMSCs cultured in FM, several lncRNAs (*AL110504.1, AL391069.1*, and *AC010273*.1) were upregulated (Fig. 4d and Additional File 3c). It would be interesting to determine the function of these lncRNAs. HP pMSCs cultured in FM had only two upregulated genes (*HIST2H3PS2 and RNH1*) associated with the DNA replication, while pMSCs cultured in XM had several upregulated genes (*RFC2, POLD2, RFC4, PCNA, FOXO1* and *RBL1*) related to the DNA replication (Fig. 4e and Additional File 3d).

pMSCs grown in FM and XM also had DEGs associated with TGFβ signaling (Fig. 4f and Additional File 3e). Several of DEGs (*TFDP1, RBL1, MYC, RPS6KB2, E2F4*, and *SP1*) and two DEGs (*CREBBP* and *EP300*) were associated with cell cycle and chromatin remodeling, respectively. These DEGs were upregulated in pMSCs grown in XM but downregulated in FM. Whereas five DEGs (*ID3, RBXI, PPP2CA, PPP2R1B*, and *PPP2CB*) involved in the negative regulation of the cell cycle were upregulated in pMSCs grown in FM. Not surprisingly, several DEGs (*SMAD7, GDF5, SMAD3, ACVR2A, SMAD2, SMAD4, TGFβ3, INHBA, BMP6, TGFβ2, TGFβRI, BMPR1A*, and *ZFYVE16*) associated with TGFβ signaling were upregulated in pMSCs grown in FM but not in XM. Additionally, pMSCs grown in the FM had more DEGs (*CXCL8, IL6, ILIA, CDKN2A*, and *CDKNIA*) involved in senescence compared to cells grown in the XM (Fig. 4g and Additional File 3f). On the other hand, DEGs related to self-renewal, such as *E2F1, CDK2, MYC, FOXO1*, and *CCND2* were upregulated in cells grown in XM.

**Figure 5:**
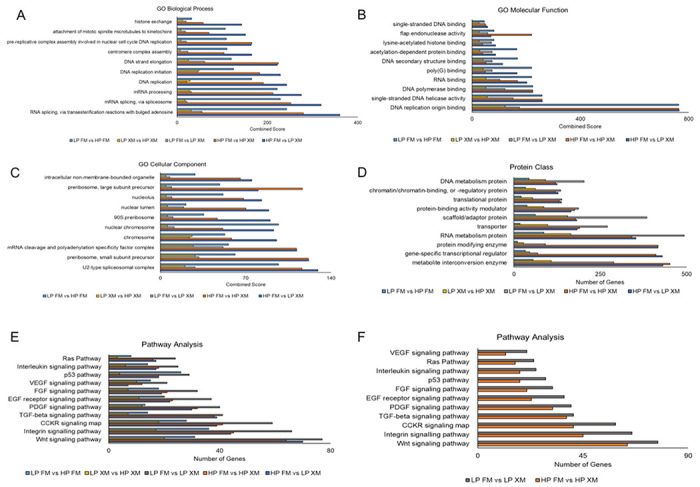
GO analysis of DEGs for LP and HP pMSCs cultured in FM and XM. (a-c) Enrichment of upregulated DEGs associated with the biological processes, molecular function, and cellular compartment, respectively, was performed with Benjamini-Hochberg FDR at p < 0.05 using DESeq2. (d-e) Upregulated DEGs associated with protein classes and signaling pathways, respectively determined by PANTHER analysis. (f) Enrichment of pathway genes when DEGs were compared between HP XM vs HP FM and LP XM vs LP FM. The Enrichr analysis generated the combined score (p-value multiplied by the z-score).

Furthermore, analysis of the DEGs, *p-value* < 0.05 using gene ontology (GO) revealed that they are associated with biological processes, molecular function, and cellular component ontologies. The most significant enrichment of biological processes such as histone exchange, attachment of mitotic spindle microtubules to kinetochore, pre-replicative complex assembly involved in nuclear cell cycle DNA replication, centromere complex assembly, DNA strand elongation, DNA replication initiation, DNA replication, mRNA processing, mRNA splicing via spliceosome, and RNA splicing via transesterification reactions with bulged adenosine as nucleophile was revealed in LP pMSCs grown in XM vs HP cells grown in FM when compared with all the groups (Fig. 5a). In contrast, a significant variation in the enrichment of molecular functions, such as single-stranded DNA binding, flap endonuclease activity, lysine-acetylated histone binding, acetylation-dependent protein binding, DNA secondary structure binding, poly(G) binding, RNA binding, DNA polymerase binding, single-stranded DNA helicase activity, and DNA replication origin binding (Fig. 5b) and cellular components such as intracellular non-membrane-bound organelle, preribosome large subunit precursor, nucleolus, nuclear lumen, 90S preribosome, nuclear chromosome, chromosome, mRNA cleavage and polyadenylation specificity factor complex, preribosome small subunit precursor, and U2-type spliceosomal complex (Fig. 5c) was observed in the cells grown in FM vs XM irrespective of the cell passage. These observations suggest that differences between molecular functions and cellular component processes could be due to the culture media.

We then subjected the RNA-seq data to PANTHER analysis, and the results are shown in Fig. 5d–f. Evidently, most proteins classes (DNA metabolism protein, chromatin/chromatin-binding or regulatory protein, translational protein, protein-binding activity modulator, scaffold/adaptor protein, transporter, and RNA metabolism protein, except protein modifying enzyme, gene-specific transcriptional regulator, and metabolite interconversion enzyme) (Fig. 5d) and signaling pathways (DNA replication, Ras, Interleukin signaling, TGFβ signaling, Cadherin signaling, p53, integrin signaling, PDGF signaling, CCKR signaling map, and Wnt signaling pathways (Fig. 5e) were enriched in LP cells grown in XM vs LP cells grown in FM when compared with all the groups. Pathway analysis comparing LP FM vs LP XM and HP FM vs HP XM revealed more enrichment in pathways such as Wnt, TGFβ, PDGF, and VEGF in the LP FM vs LP XM group (Fig. 5f)

### Interactome Analysis

Interactome analysis using GOnet showed upregulation of selected DEGs associated with cell cycle and DNA replication positive interaction between the LP and HP cells grown in XM but not in FM (Additional File 4a, b). The selected upregulated DEGs associated with proliferation showed a positive interaction among the genes expressed in LP and HP cells grown in XM but not FM (Additional File 4c, d). In contrast, selected DEGs associated with senescence revealed positive interaction among the genes expressed in LP and HP cells grown in FM but not in XM (Additional File 4e, f).

### Analysis of nucleosome occupancy

**Figure 6:**
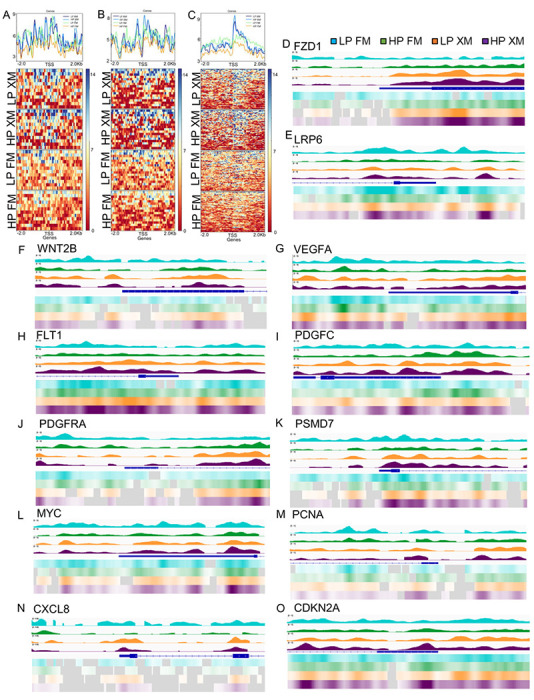
Nucleosome mapping and genome-browser shots showing nucleosome occupancy determined by Mnase-seq and IGV analysis (a-c) Nucleosome mapping showing cell cycle, DNA replication, and senescence genes, respectively. (d-f) WNT pathway genes (FZD1, LRP6, and WNT2B), (g-j) VEGF/PDGF pathway genes (VEGFA, FLT1, PDGFC and PDGFRA), (k) cell cycle gene (PSMD7), L-M Self-renewal and DNA replication genes (MYC and PCNA), and (n-o) senescence (CXCL8 and CDKN2A). Data showed less nucleosome occupancies in pMSCs cultured in XM compared to FM in cell cycle and DNA replication genes. In contrast, more nucleosome occupancies in pMSCs cultured in XM compared to FM was found in genes involved in senescence. IGV mapping showed that FZD1, LRP6, WNT2B, VEGFA, FLT1, PDGFC, PDGFRA, PSMD7, MYC, and PCNA genes had less nucleosome occupancies around the promoters, suggesting the higher expression in pMSCs grown in XM than FM. Whereas CXCL8 and CDKN2A had higher nucleosome occupancies around the promoters, suggesting their lower expression in pMSCs grown in XM compared to FM. LP and HP pMSCs grown in FM (blue and green, respectively), LP and HP pMSCs grown in XM (orange and purple, respectively).

In addition, we performed the MNase-seq analysis of pMSCs cultured in the two different media (Fig. 6a–c). These results showed more nucleosome occupancy in LP and HP pMSCs cultured in the FM compared to LP pMSCs grown in the XM in genes involved in some cellular functions, particularly cell cycle and DNA replication (Fig. 6a, b respectively). On the other hand, less nucleosome occupancy was observed in HP pMSCs cultured in the FM compared to the cells grown XM in genes involved with the senescence pathway (Fig. 6c). This suggests that genes related to cell cycle and DNA replication in pMSCs cultured in XM, and genes related to senescence have lower histone occupancies that could promote higher expression in HP pMSCs cultured in FM.

### Integrative Genomics Viewer (IGV) Mapping

Since RNA-seq analysis revealed passaging and medium composition affected the expression, we examined MNase-seq data to evaluate nucleosome occupancies of selected genes involved in selfrenewal, cell cycle, DNA replication, differentiation, and senescence. IGV maps show less nucleosome occupancies in the area around the promoters of genes associated with Wnt (*FZD1, LRP6, and WNT2B*), VEGF/PDGF (*VEGFA, FIJI, PDGFC*, and *PDGFRA*), cell cycle (PSMD7), and self-renewal (MYC), and DNA replication (PCNA) (Fig. 6d–m, respectively) in pMSCs cultured in XM compared to FM. In contrast, senescence genes (CXCL8 and CDKN2A) had less nucleosome occupancy in pMSCs cultured in FM than XM (Fig. 6n–o). These results suggest that upregulation of the self-renewal and proliferation genes show lower nucleosome occupancies in cells grown in XM consistent with their higher expression. In contrast, senescence genes are induced in cells grown in FM, consistent with their higher expression.

### Expression of selected genes

**Figure 7:**
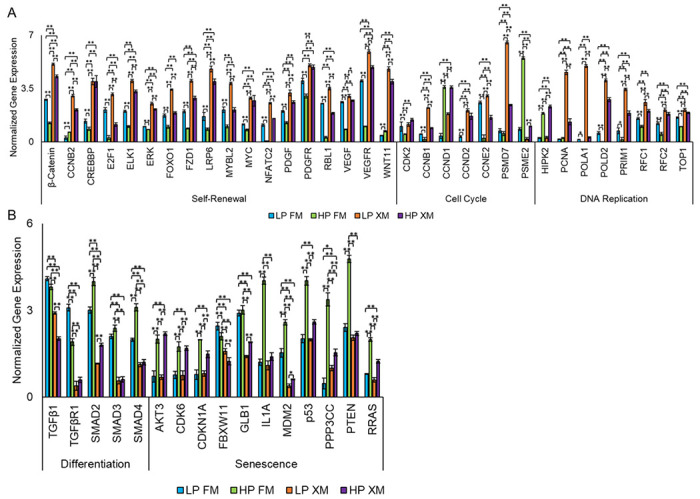
Relative expression of genes in pMSCs cultured in FM and XM. (a) Genes involved in self-renewal, cell cycle, and DNA replication. (b) Genes involved in differentiation and senescence. Gene expression was normalized to GAPDH and ACTIN, and error bars represent the standard deviations of the triplicate measurements.

Finally, we validated the expression of selected DEGs by qRT-PCR. The results depicted in Fig. 7a, show self-renewal genes (*β-catenin, CCNB2, CREBBRE2F1, ELK1, ERK, FOXO1, FZD1, LRP6, MYBL2, NFATC2, PDGF, PDGFR, RBL1, VEGF, VEGFR*, and *WNT11*) upregulated in pMSCs cultured in XM. In addition, several cell cycle genes (*CCNB1, CCND2, CCNE2*, and *PSMD7*) were upregulated in LP pMSCs grown in XM. Only one cell cycle gene was upregulated in HP pMSCs cultured in FM and XM (*CCND1*), and only *PSME2* was upregulated in HP cells grown in FM. Several genes related to DNA replication (*PCNA, POLA1, POLD2, PRIM1, RFC1, RFC2*, and *TOP1*) were upregulated in pMSCs grown in XM compared to FM. Only one DNA replication gene (*HIPK2*) was upregulated in HP pMSCs grown in FM, but its significance remains to be investigated. In contrast, several differentiation genes (*TGFβ1, TGFβR1, SMAD2, SMAD3*, and *SMAD4*) were upregulated in pMSCs cultured in FM (Fig. 7b) than XM. Furthermore, we found several senescence genes (*CDKN1A, GLB1, IL1A, MDM2, p53, PPP3CC, PTEN*, and *RRAS*) highly upregulated in HP pMSCs cultured in FM. These results suggest XM supported self-renewal and FM induced differentiation and senescence in pMSCs.

## Discussion

Although MSCs can be isolated from various sources [1–3], they display variable properties, particularly self-renewal, differentiation, and anti-inflammatory and immunomodulatory capabilities (3, 4, 31, 32). In addition, their properties change during propagation depending on the culture conditions (15–19, 23). Many studies have reported that MSCs lose their self-renewal or proliferation ability upon passaging (24, 25). Maintenance of MSC properties during propagation is one of the major obstacles to obtaining reproducible and consistent results in clinical applications. As a result, the widespread therapeutic use of MSCs is hampered due to the inconsistencies in their properties.

This study investigated the changes in pMSCs grown in FM and XM. First, we investigated the morphology and cell surface markers of pMSCs. The cells grown in FM displayed elongated and larger cell morphology compared to XM. However, pMSCs cultured in both media had similar expression of cell surface markers (CD29, CD44, CD73, and CD105) except CD90 and ITGA/CD49f, markedly reduced in FM. Studies have shown that CD90 level is decreased upon differentiation of MSCs (33, 34). In contrast, CD49f is a self-renewal marker expressed in over 30 types of stem cells (35, 36).

Further analysis of pMSCs in FM showed a rapid increase in DT from 25 h at P3 to over 50 h at > P25. In contrast, the DT for cells grown in XM remained steady and slowly increased from 15 h at P3 to 27 h at P40. Several published studies showed a significant increase in DT of adult and umbilical cord-derived MSCs upon passaging in FM compared to XM (20–22). In addition, the CFE of pMSCs was significantly lower in FM compared to XM. The highest CFE and more compact colonies were observed in LP cells grown in XM. The BrdU assay also revealed reduced proliferation of pMSCs grown in FM. Telomerase activity, a self-renewal marker, has been shown to decrease when stem cells gradually differentiate or enter into senescence (37–39). Adult MSCs have relatively low or no telomerase activity (39–41). Analysis of pMSCs showed a lower level of telomerase activity than hESCs and HEK cells. Relative telomerase activity remained almost the same from P3 to 20 in the XM, but it was lost in FM when grown to HP.

We wondered if the media affected the differentiation potential of pMSCs as well. Therefore, we examined the trilineage differentiation of pMSCs grown in FM and XM. Although pMSCs cultured in either media differentiated into adipogenic, chondrogenic and osteogenic lineages, there were significant differences. HP pMSCs grown in FM showed a greater tendency to differentiate towards adipogenic lineage. pMSCs grown in XM and LP cells grown in FM showed a higher differentiation tendency towards chondrogenic and osteogenic lineage. Interestingly, MSCs have been shown to lose their self-renewal and trilineage differentiation potential but still be able to differentiate towards the adipogenic lineage (42–45).

Although several studies have shown a successive decrease in the self-renewal and differentiation potential, molecular changes responsible for this shift in the properties/characteristic of MSCs have been poorly investigated (46, 47). We envision that pMSCs also undergo molecular level changes, including cell signaling pathways dictating changes in their characteristics. To address this, we performed transcriptomic and MNase-seq analyses of pMSCs. The results showed significant differences in the gene expression in pMSCs grown in the two media. However, there was little difference between LP and HP cells. Expectedly, volcano plot analysis of DEGs in pMSCs also showed greater DEGs when comparing the two media. However, only slight differences in DEGs were found between the LP and HP cells. Altogether, these results suggest that medium composition imparted a greater influence on the fate of pMSCs.

WNT signaling plays an important role in self-renewal and differentiation in various stem cells, including MSCs (48,49). As expected, XM-grown pMSCs had upregulated expression of Wnt genes (*AXIN1, CREBBP, CSKNK1G2, DVL1, DVL2, FZD1, MYC, PRKCD, SRCAR and TLE3*) related to positive regulation of selfrenewal. In contrast, pMSCs cultured in FM had upregulated in Wnt genes (*CCND1, PPP2B, PPP2CA, PPP2R5B, SFRP1, TCF7L1*, and *TLE4*) related to negative regulation of self-renewal or induction of differentiation. In addition, cells grown in XM also had upregulation of two Notch signaling genes, *SRCAP* and *TLE3*, which are suggested to regulate self-renewal capacity and multipotency of stem cells (50, 51). More over, several members of SNF/SWI gene family (*SMARCA2, SMARCA4, SMARCC2, and SMARCD1*) were upregulated in pMSCs cultured in XM. In contrast, pMSCs grown in FM had upregulated expression of genes (*TGFBR1* and *BMPR1A*) that promote differentiation (52, 53). In addition, *SMARCA1/SNF2L*, a chromatin remodeling ATPase involved in DNA damage response (54), was upregulated in cells grown in FM. Several DEGs (*CDH4, CDHR2, CELSR3, PCDHGA10, PCDHGA12, PCDHW, DCHS1, PCDH9, PCDH7, PCDHGC5, PCDHGC3, PCDHW, PCDHGB3, PCDHGA6, CELSR2, CDH11, CDH2, PCDHGB4, PCDHGA7, PCDHGA2*, and *FAT4*) of cadherin superfamily were found in pMSCs cultured in both FM and XM. Cadherin plays a role in cell-to-cell adhesion (55). These results suggest that XM but not FM supported the maintenance of self-renewal.

Cell proliferation is regulated by the interactions of Cyclin/CDK and Myc/E2F (56–59). During the G1 phase of the cell cycle, CCND complexes with *CDK4*, which causes the *RB* to be phosphorylated. This phosphorylation upregulates *CCNE* with *CDK2* transcription and causes the cells to enter the S phase(57, 60). *MYC* also helps mediate the *E2F* promotion of the cells transiting from the G1 phase to the S phase (56, 57). Our data showed that *E2F1* and *CDK2* were upregulated in cells cultured in XM and LP cells cultured in FM. Whereas several genes related to the progression of proliferation (CCND2, CCND3, CCNE2, CDK2, PCNA, POLA1, POLD2, PRIM1, RFC1, RFC2, and RFC4) were upregulated in pMSCs cultured in XM. These genes were downregulated in pMSCs cultured in FM. Interestingly several IncRNA genes (*AC010273.1, AL391069.1*, and *AL110504.1*) were upregulated in cells grown in FM. lncRNAs are important epigenetic regulators at transcriptional and translational levels (61). Further studies are warranted to understand the induction and role of these lncRNAs in determining the fate of pMSCs.

We found that many genes (*RBL1, MYC*, and *E2F4*) of the TGFβ pathway were upregulated in XM but downregulated in FM-grown pMSCs. Interestingly these genes are involved in the positive regulation of self-renewal (62). Furthermore, two genes (*EP300* and *CREBBP*) that regulate gene expression by intrinsic histone acetylation activity (63, 64) were also upregulated in cells grown in XM. Several genes (*SMAD2 SMAD3, SMAD4, SMAD7, TGFβR1, TGFβ2, TGFβ3, BMPR1A*, and *BMP6*) of TGFβ pathway were also upregulated in FM-grown pMSCs. These genes are known to play a role in inducing differentiation (53). In addition, three genes (*PPP2CA, PPP2R1B*, and *PPP2CB*) of TGFβ pathway that negatively regulate cell cycle by dephosphorylating RB (65, 66) were upregulated in cells grown in FM. These findings concur with the report suggesting the involvement of the TGFβ signaling pathway in regulating diverse cellular processes, including proliferation and differentiation (67). Since FM-grown cells had progressively increased DT and reduced CFE, and downregulated self-renewal genes, we wondered if these changes further impacted the fate of pMSCs towards senescence.

Senescence is an important contributor to aging and cancers, two processes characterized by a time-dependent accumulation of cell damage and dysfunction. Senescent cell growth arrested in the G1 phase prevents DNA replication initiation in damaged cells (68, 69) and in G2 to block mitosis in the presence of DNA damage (70). It is well established that MSCs progressively undergo senescence upon passaging (71–75). The transcriptional analysis also revealed that the total number of senescence genes was highest in HP pMSCs grown in FM. On the other hand, DEGs found in LP, and HP pMSCs grown in XM were predominantly inhibitory to senescence. pMSCs grown in XM had upregulated expression of genes (*MYC, CCND2, CCND3, MYBL2*, and *E2F4*) related to positive regulation of self-renewal. Whereas the DEGs (*SMAD3, TGFβRT, TGFβ2*, and TGF*β*3) in pMSCs grown in FM are involved in the TGFβ signaling pathway as discussed above. A senescence marker, *GLB1* (SA-B gal) was only upregulated in HP pMSCs grown in FM. This was consistent with a previous report where adipose-derived MSCs showed an increase of SA-B gal-positive cells upon passaging (76).

Furthermore, *CHEK2* was upregulated in pMSCs grown in FM. *CHEK2* phosphorylates *p53*, which activates *CDKN1A* (p21) and inhibits CDK-cyclin complexes such as *CDK2*(77, 78). *CDKN1A* (p21) was upregulated, and *CDK2* was downregulated in HP pMSCs cultured in FM, causing cell cycle arrest. GO analysis of DEGs in pMSCs grown in two different media revealed a clear difference in the enrichment of biological processes, molecular function, and cellular components. However, there was little difference between the LP and HP pMSCs. PANTHER analysis of the DEGs in pMSCs also showed a similar trend regarding the enrichment of protein class and signaling pathways. Many enriched signaling pathways regulate self-renewal, cell cycle, differentiation, and senescence. Pathway analysis comparing LP FM vs LP XM and HP FM vs HP XM revealed more enrichment in WNT, TGFβ, PDGF, and VEGF pathways in the LP FM vs LP XM group. GOnet analysis showed that selected upregulated cell cycle, DNA replication, and proliferation genes show positive interaction in pMSCs grown in XM but not in FM. In comparison, selected senescence genes had positive interaction among the genes expressed in pMSCs grown in FM, but not in XM.

To further examine these results at a chromatin level, we performed MNase-seq analysis. We determined that cell cycle and DNA replication genes had more nucleosome occupancy in pMSCs cultured in the FM compared to the XM. In contrast, less nucleosome occupancy was observed in senescence genes in HP pMSCs cultured in the FM but not in the XM. Our results showed that cell cycle and DNA replication genes were upregulated in XM-grown cells, and on the other hand, senescence genes were upregulated in FM-grown cells. The lower nucleosome occupancies at these genes is consistent with upregulation.

We evaluated the MNase-seq data using IGV. IGV mapping revealed low nucleosome occupancy in the area surrounding promoters of genes related to WNT and VEGF/PDGF signaling pathways. In addition, genes associated with cell cycle, self-renewal, and DNA replication also had low nucleosome occupancy in XM-grown pMSCs. Whereas the selected senescence DEGs had less nucleosome occupancy in FM-grown pMSCs, consistent with their higher expression.

The expression of DEGs was also validated by qRT-PCR. The selected DEGs involved in self-renewal (*β-catenin, CCNB2, CREBBP, E2F1, ELK1, ERK, FOXO1, FZD1, LRP6, MYBL2, NFATC2, PDGF, PDGFR RBL1, VEGF, VEGFR*, and *WNT11*), cell cycle (PSMD7, CCNE2, CCND2, CCNB1, and CDK2), and DNA replication (POLA1, PCNA, *POLD2, PRIM1, RFC1, RFC2*, and *TOP1*) were highly expressed in cells grown in XM. On the other hand, DEGs involved in TGFβ (TGFβ1, TGFβRI, SMAD2, SMAD3, and SMAD4) and senescence (*CDKN1A, GLB1, IL1A, MDM2, p53, PPP3CC, PTEN*, and *RRAS*) DEGs were more expressed in pMSCs grown in FM.

**Figure 8:**
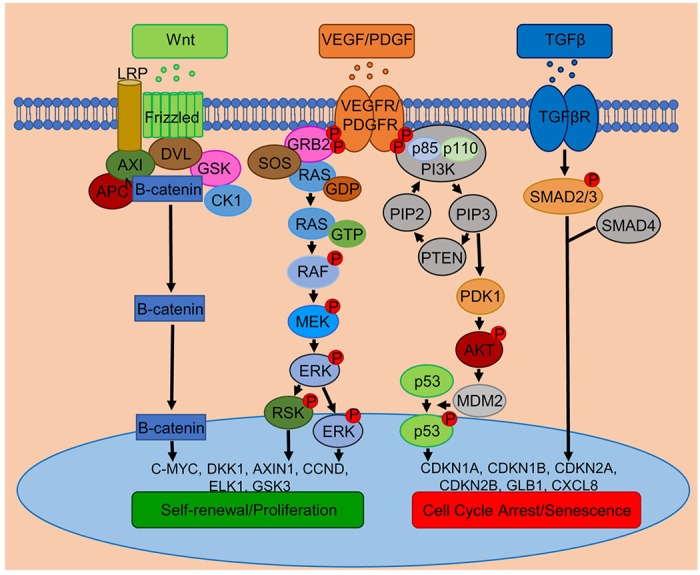
Proposed WNT, VEGF/PDGF, and TGF signaling pathways mediating self-renewal and cell cycle arrest in pMSCs.

Based on the accumulated evidence, we propose a mechanism of self-renewal or cell arrest, as depicted in Fig. 8. In XM, the WNT protein keeps the WNT pathway active by binding to the Frizzled receptor, and the destruction complex (DVL/AXIN/APC/GSK3*β*/CK1) remains destabilized and thus allowing the *β*-catenin to promote the expression of self-renewal genes in the nucleus. In addition, growth factors, VEGF and PDGF play a role in the proliferation of pMSCs in XM by phosphorylating the respective receptors, which recruit the adaptor protein GRB2, and the nucleotide exchange factor SOS, which sequentially activates RAS, RAF, MEK, and ERK. The phosphorylated ERK then enters the nucleus and activates the transcription of cell proliferation genes like c-MYC. Alternatively, ERK activates RSK, which then activates proliferation genes. On the other hand, in FM, PI3K is activated with an increase in the expression of PTEN and PDK1, which activate the AKT. Presumably, activated AKT phosphorylates MDM2 to modulate the p53 activity responsible for senescence gene expression. Senescence is also gradually induced by the interaction of TGFβ with TGFβR, that activates SMAD2/3 by phosphorylation and moves to the nucleus with SMAD4 to turn on genes involved in cell cycle arrest.

## Conclusion

In conclusion, the results show that XM is more efficient than FM in maintaining the self-renewal and differentiation potential of pMSCs. In FM, pMSCs gradually change morphologically and the trilineage differentiation capacity. In addition, they have reduced proliferation and CFE. Transcriptomic analysis suggests WNT and VEGF/PDGF signaling may be involved in maintaining the self-renewal and proliferation of pMSCs. However, TGFβ and PI3K signaling may be responsible for gradual self-renewa loss and senescence induction. Our findings may help expand pMSCs for large clinical studies to treat degenerative diseases.

## Data Availability

The raw and processed sequencing data generated in this study have been deposited in the NCBI GenBank database, accession code PRJNA928451. All data needed to evaluate the conclusions are present in the paper and/or the Supplementary Materials.

## Abbreviations

CFE: Colony forming efficiency
CD: Cluster of differentiated
DEGs: Differentially expressed genes
DT: Doubling time
FM: Fetal bovine serum medium
HEK: Human embryonic kidney cells
hESCs: Human embryonic stem cells
HP: High passage
LP: Low passage
HP FM: HP pMSCs grown in FM
HP XM: HP pMSCs grown in XM
LP FM: LP pMSCs grown in FM
LP XM: LP pMSCs grown in XM
MNase-seq: Micrococcal Nuclease Sequencing
MSCs: Mesenchymal stem cells
P: Passage
p: Primitive
qRT-PCR: Quantitative reverse transcriptase polymerase chain reaction
RNA-seq: RNA-Sequencing
SEM: Standard error of the mean
XM: Xeno-free medium
